# Sex specific seasonal variation in the diet of brown bears in human dominated landscapes

**DOI:** 10.1038/s41598-025-21232-x

**Published:** 2025-10-27

**Authors:** Maura Francioni, Alexandra Veselovská, Nuno F. Guimarães, Peter Klinga, Rudolf Kropil, Ladislav Paule, Peter Smolko

**Affiliations:** 1 Department of Applied Zoology and Wildlife Management, Faculty of Forestry, Technical University in Zvolen, T. G. Masaryka 24, 960 01 Zvolen, Slovakia; 2 Plant Diversity and Ecosystems Management Unit, School of Biosciences and Veterinary Medicine, University of Camerino, Camerino, MC Italy; 3 State Nature Conservancy of the Slovak Republic, Tajovského 28B, 974 01 Banská Bystrica, Slovakia; 4 Department of Phytology, Faculty of Forestry, Technical University in Zvolen, T. G. Masaryka 24, 960 01 Zvolen, Slovakia

**Keywords:** Diet composition, Trophic ecology, Sex-specific diet, Anthropogenic influence, Large carnivore, Slovakia, Ecology, Zoology

## Abstract

Understanding the foraging ecology of brown bears (*Ursus arctos*) in human-dominated landscapes is essential for effective wildlife management and conflict mitigation. In this study, we investigated the seasonal diet composition of brown bears in the Western Carpathians, Slovakia, using a combination of molecular genetic and microhistological scat analyses. We analysed 198 brown bear scats (101 males, 97 females) collected throughout the year in central Slovakia, to assess seasonal variation in diet, identify sex-specific foraging patterns, and evaluate the use of anthropogenic food resources. Vegetation constituted the primary dietary component year-round, with hard mast frequency dominating in spring (*FO* = 45%), autumn (*FO* = 67%), and winter (*FO* = 82%), while soft mast (*FO* = 48%) and anthropogenic food (*FO* = 30%) prevailed in summer. Dietary energy intake from anthropogenic food was considerable in spring (*EDEC* = 15%) but peaked in summer (*EDEC* = 34%) and was consistently higher in males across all seasons. In spring, males consumed a considerable amount of anthropogenic food (*EDEC* = 32%), while it was negligible for females (*EDEC* = 3%). In summer, male consumption of anthropogenic food increased further (EDEC = 40%), and although females still consumed less than males, their intake rose by 23% compared to spring (*EDEC* = 26%). Males also consumed more vertebrates and hard mast, whereas females relied more heavily on soft mast and natural food sources, likely reflecting sex-specific trade-offs between energetic needs and risk avoidance. Seasonal dietary diversity was highest in summer and lowest in winter, with the greatest trophic divergence between sexes occurring during spring and summer. These findings highlight the dietary plasticity of brown bears in response to seasonal and anthropogenic resource availability, and emphasize the importance of regulating anthropogenic food access, particularly supplemental feeding and agricultural crops, to reduce human-bear conflict.

**Supplementary Information:**

The online version contains supplementary material available at 10.1038/s41598-025-21232-x.

## Introduction

Studying the foraging behaviour of wild animals is essential for understanding their ecological niches and identifying key environmental resources, both of which are critical for developing effective management strategies for species and ecosystems^1,2^. This is particularly important for generalist omnivores with broad geographic ranges, such as the brown bear (*Ursus arctos*), whose diet exhibits considerable regional differences. For instance, brown bears display predominantly herbivorous diets in southern^3,4^ and central Europe^5,6^ but shift to more carnivorous diets with latitude^7^, i.e., Siberia^8^, Scandinavia^9,10^ and North America^11^. Facing the increase in brown bear population throughout Europe over the last two decades^12^, it is crucial to fully understand their foraging habits and ecological adaptations in human-modified landscapes because they have direct influence on the type and extent of bear-human conflicts^13^.

In temperate forests of the Slovak Carpathians, vegetation generally constitutes the primary component of the brown bear diet, with the availability of hard and soft mast being particularly important^5,6^. Vertebrates and insects, as sources of high-quality animal protein, also regularly contribute to the diet, especially in spring and summer^6^. However, bear diet composition is primarily driven by the availability and quality of foraging resources and their spatial and temporal dynamics^10,14,15^. For example, high-quality forage resources are often patchily distributed across the landscape and fluctuate over time due to seasonal changes, natural depletion from use, or anthropogenic alterations^4,6,15^. Anthropogenic foods are often exploited by brown bears when available due to their high-energy yield and low foraging cost, creating a significant potential to influence bear behaviour, physiology, and demography^16–18^. In Slovakia, bears generally obtain anthropogenic food through two primary sources: first, by utilizing food intended for the supplementary feeding of wild ungulates provided by hunters during winter and early spring^5^; and second, by capitalizing on agricultural practices in summer, such as large-scale crop cultivation, which provide nutrient-rich, easily accessible food sources^6^.

In addition to external factors, the social structure of a bear population can significantly influence dietary patterns through despotism, where dominant individuals displace others from high-quality habitats^19^. Additionally, females with cubs avoid male territories to reduce the risk of infanticide, as dominant males may kill unrelated young to increase their mating opportunities^19^. Together, these factors create a strong hierarchy within bear populations, resulting in spatiotemporal segregation, differential habitat use, and unequal access to high-quality resources among individuals^19–21^. Physiological requirements based on body size also significantly shape dietary patterns. For instance, females with cubs, who have lower absolute energy needs than larger males, are more efficient at exploiting invertebrates such as ants and other insects, while also minimizing the infanticide risk^3^. In contrast, males tend to consume higher proportions of vertebrates, reflecting their greater energy demands^21,22^.

In this study, we examine the seasonal trophic ecology of brown bears in the Western Carpathians, Slovakia, using a combination of molecular genetic techniques and traditional microhistological analysis, with a focus on dietary differences between male and female bears. Genetic analysis eliminates scat misidentification with other species and provides information on individual characteristics such as sex, which are rarely known from faecal samples^19^. Specifically, our objectives were: (1) to document the seasonal dietary habits of brown bears inhabiting a human-dominated landscape of the Western Carpathians, (2) to analyze differences in trophic strategies of male and female brown bears, and (3) to evaluate the use of anthropogenic food by bears and propose implications for management practices. We hypothesize that vegetation will be the primary component of the brown bear diet throughout the year in our study area^5,6^. We also expect to find significant differences in dietary composition between males and females, especially regarding highly nutritious food types including anthropogenic food^19,21,22^.

## Methods

### Study area

**Fig. 1:**
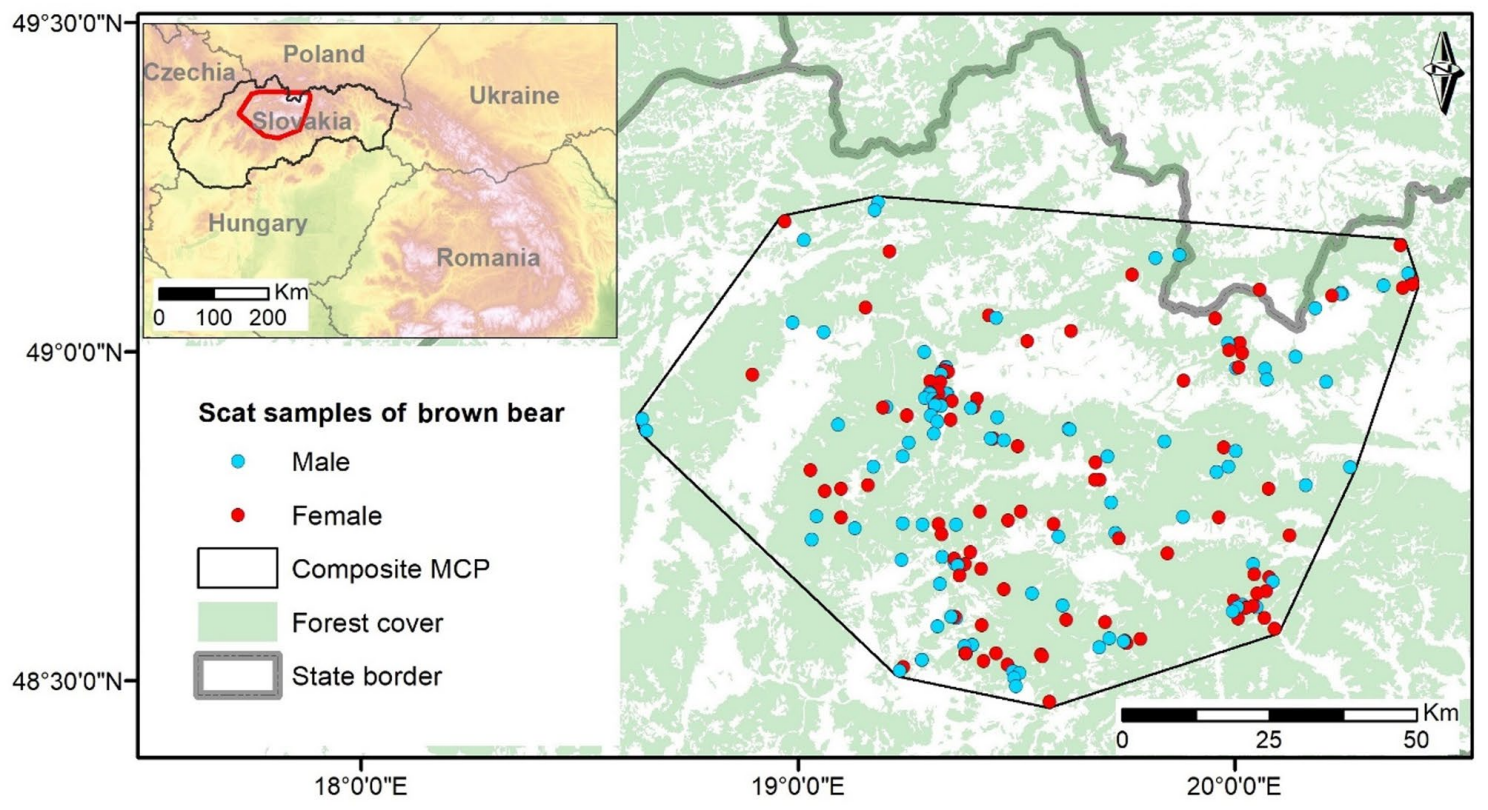
Study area and brown bear scat sample locations (*n* = 198) in the Western Carpathians, Slovakia, during 2013–2014. All base map layers, including elevation and administrative boundaries, were obtained from the Slovak Geoportal (https://www.geoportal.sk; CC BY 4.0 license, Institute of Geodesy and Cartography Bratislava, 2022). Forest cover from CORINE Land Cover 2018 (European Environment Agency). Map prepared in ArcMap 10.6 (Esri, Redlands, CA, USA).

The study was conducted in the Western Carpathian Mountains, Slovakia, covering an area of 7 873 km^2^ and encompassing approximately 48% of the brown bear range in Slovakia (Fig. 1). The area had an elevation range of 355–1871 m a.s.l., the mean annual precipitation was 1139 mm, and the mean annual temperature was 6 °C. The landscape consisted of 28.5% coniferous forest, 19.0% mixed forest, and 10.0% broad-leaved forest, resulting in a total forest cover of 57.5%. Additionally, 10.0% of the area was shrubland, 28.0% is agricultural land, 4.0% was occupied by human structures, 1.5% was rocky terrain with sparse or no vegetation, and 0.4% was water bodies^23^. Deciduous forests are primarily composed of European beech (*Fagus sylvatica*), with a mix of several oak species (*Quercus* spp.) with European hornbeam (*Carpinus betulus*), and presence of European red raspberry (*Rubus idaeus*) and bramble (*Rubus* spp.). Coniferous forests consist mainly of Norway spruce (*Picea abies*), which naturally dominates elevations above 1000 m a.s.l., with mixture of silver fir (*Abies alba*), Scots pine (*Pinus sylvestris*), and European larch (*Larix decidua*), and blueberry (*Vaccinium myrtillus*) and cranberry (*Vaccinium vitis-idaea*) at ground level. Shrub layer around forest edges often includes fruit-bearing species such as dog rose (*Rosa canina*) and blackthorn (*Prunus spinosa*).

Alongside the gray wolf (*Canis lupus*) and Eurasian lynx (*Lynx lynx*), the brown bear was the most common large carnivore in the region, with an estimated density of 10.0 individuals per 100 km^2^ in 2014^24^. The growth of Slovakia’s brown bear population has been largely attributed to legal protections under national and EU legislation, which recognize the species under Annex II and Annex IV of the EU Habitats.

### Scat collection

This study was based exclusively on non-invasive sampling of brown bear scats collected in the field. No animals were captured, handled, or otherwise disturbed for the purposes of this research; therefore, formal ethical approval was not required. All methods were carried out in accordance with relevant guidelines and regulations, and the study is reported in accordance with the ARRIVE guidelines (https://arriveguidelines.org). Fresh brown bear scats were collected year-round within the bears’ stronghold in central Slovakia (Fig. 1) during 2013–2014 within the project “Research and monitoring of large carnivores and wildcat populations in Slovakia”^24^. Scats were found opportunistically during weakly extensive surveys conducted in all types of habitats according to their availability. All collected scat samples of brown bear within the project (*n* = 3155) were stored in 96% ethanol and subsequently genotyped. Out of the 1834 successfully genotyped scat samples, we randomly selected 198 samples (9.26%) stratified by season and sex (Table 1) for the microhistological analysis of the bear diet (average dry weight = 1.2 ± 0.1 g; mean ± SE).

### Genetic analysis

DNA was extracted using the DNA Stool Mini Kit (Qiagen, Hilden, Germany). We identified individual genotypes amplified by microsatellite markers: Mu10, Mu50, Mu59^25^, G10L, G1D^26^, G10P, G10C^27^, Mu15. Sex of individuals was determined using ZFX/Y and SRY markers^28^. Negative and positive controls were ensured across all reactions. PCR reactions and DNA amplifications of samples were conducted using the same procedure^29^. Allele sizes were determined using the GeneMapper 3.7 software^30^. Each sample was amplified at least three times to obtain reliable consensus on genotypes^31,32^. Genotypes were tested for allelic dropout and null alleles using Microchecker software^33^. Identical multilocus genotypes were identified with samples amplified at a minimum of seven loci^34^ and duplicate genotypes that differed in two or fewer alleles were omitted from further analyses^24^.

### Seasonality

We categorized selected scat samples into four seasons (Table 1) reflecting physiological stages and feeding behaviour of brown bears^5,6^ but also forage availability throughout the year: spring (March–May) corresponding to hypophagia (high availability of young grasses, forbs and germinating seeds, limited availability of insects), summer (June–August) corresponding to early hyperphagia (high availability of forest fruits, agricultural crops and insects), autumn (September–November) corresponding to late hyperphagia (high availability of beechnuts, acorns and other seeds and forest fruits, limited availability of insect), and winter (December–February) corresponding to hibernation period (limited availability of beechnuts, acorns and other seeds, occasional anthropogenic food from supplementary winter feeding).

**Table 1:** Number of male and female brown bear scat samples (*n* = 198) collected in Slovakia during 2013–2014 used in our dietary analysis.

Sex	Season			
	Spring	Summer	Autumn	Winter
Male	28	30	30	13
Female	28	30	30	9
Sum	56	60	60	22

### Diet analysis

To quantify the seasonal diet of brown bear males and females, we used microhistological analysis of scat fragments. Samples were placed in Petri dishes and dried in the oven at 68 °C for 12 h. Each sample was weighed and washed through a sieve of 0.8 mm until the water was clear. We repeated the drying process before proceeding to further analyses. Cleaned samples were evenly spread on a sheet. First, all undigested fragments were categorized into four groups, i.e., plants, invertebrates, vertebrates, and others (gravel, wood and bark fragments, needles, unidentifiable items). Second, we excluded the category “others” from further analyses, since it likely represents contaminants or consumed items with no nutritional value for brown bear as a food source^9^ and only plants, invertebrates and vertebrates were used for further analyses. Third, because plants make up the majority of the brown bear diet^4,6^ we used a microhistological key^35^ to further identify semi digested plant fragments into more detailed categories such as hard mast (mainly acorns and beechnuts), soft mast (flashy fruits and berries), grasses and sedges, herbaceous and tree leaves, and agricultural crops (wheat, corn, oats) considered as anthropogenic food (Table S1). As a result, we ended up with seven food categories, i.e. the five plant categories, invertebrates and vertebrates. The volumetric proportion of each food category within the scat was estimated by the point frame method^36,37^. Identification of vertebrate species was based on morphologic features of hairs found in scats, such as cuticular, medulla, and cross-section patterns, using available hair identification keys^38,39^. We removed all non-prey vertebrate species, such as bear (*n* = 18), wolf (*n* = 4), and fox (*n* = 2), from further analyses, as their presence may result from bears licking themselves or scavenging from other predators’ kills. Thus, we considered only wild ungulates and rodents as typical prey species for bears^21^. Invertebrates were identified to the order (*Hymenoptera*, *Coleoptera*) and if possible, also to the family level (e.g., *Formicidae*, *Apidae*, *Vespidae*) and their developmental stage (e.g., eggs, larvae) based on their morphologic features^40^.

Next, we calculated the frequency of occurrence (hereafter FO) and the faecal volume (hereafter FV) of each food category per male and female in all four seasons as: *FO*% = (*N*_*i*_ ÷ *N*_*t*_) × 100, where *N*_*i*_ is the number of occurrences of food item “*i*” and *N*_*t*_ is the total number of samples; resp. *FV*% = (*V*_*i*_ ÷ *V*_*t*_) × 100, where *V*_*i*_ is the volume of food item “*i*” and *V*_*t*_ is the total volume of all food items. Because FO and FV tend to underestimate the contribution of highly digestible and energy-rich foods^42^, we used correction factors^41,42^ to estimate the dietary content (hereafter EDC%) which represents the original diet composition, and the dietary energy content (hereafter EDEC%) which represents the digestible energy a bear obtains from ingested food (Hewitt and Robbins 1996; Table S1). We calculated EDC as: *EDC*_*i*_% = (CF1_*i*_ × FV_*i*_) ÷ Σ(CF*1*_*j*_ × FV_*j*_) × 100, where CF_*i*_ is the correction factor for category “*i*”, FV_*i*_ is the faecal volume for category “*i*”, and “*j*” represents all food categories included in the analysis; resp. *EDEC*_*i*_% = (CF2_*i*_ × *EDC*_*i*_) ÷ Σ(CF2_*j*_ × *EDC*_*j*_) ×100. We considered estimates of EDEC to be the most important measure, as the energy contribution of a food item is assumed to most accurately reflect its true importance to bears^9^.

To evaluate the diversity of the seasonal bear diet (niche breadth), we calculated Shannon-Wiener diversity index (*H*’)^43^, and to evaluate seasonal niche overlap between males and females, we calculated Pianka’s index of niche overlap (*O*)^44^.

### Statistical analyses

To examine seasonal differences in the FO of individual food items in the diet of brown bears, we fit a binomial generalized linear mixed-effect model (GLMM) with item occurrence as the response variable, season as the predictor variable and animal ID as random effect. We used the *glm* function in R with a binomial error structure and logit link function. Following the GLMM, we performed pairwise post-hoc comparisons to assess significant differences between seasons. Given the binomial nature of the response, we employed the *glht* function from the *multcomp* package in R, which provides adjusted p-values for multiple comparisons based on Tukey’s method. All analyses were conducted in R (version 4.1.3)^45^. To examine seasonal differences in the FV of individual food items by brown bears, we used beta regression analysis, using the *betareg* package, with item volume as the response variable and season as the predictor. We obtained estimated marginal means for each season using the *emmeans* package and conducted pairwise comparisons adjusted by Sidak’s correction. To assess seasonal differences in EDC and EDEC%, pairs of seasons were compared using two-proportion Z tests with continuity correction^46^. To provide a clear visual representation of the results, significance letters were generated using the *multcompView* package, which facilitated the identification of groups with statistically similar or different dietary compositions. For assessing differences between male and female brown bears, we applied a two-proportion Z test. The diversity of the diet was analysed using linear mixed effects (LME) models, with sex, season, and their interaction used as fixed effects, and animal ID as a random effect. Significant differences in diet diversity were identified using Tukey’s HSD test. For clarity, we report only statistically significant results (*P* ≤ 0.05).

## Results

We analysed 198 bear scats (Table 1) of which 101 were from males and 97 were from females (male : female = 1.04 : 1). The dataset included 169 genetically unique individuals, of which 83 were males and 86 were females (male : female = 0.97 : 1).

### Seasonal diet composition

**Fig. 2:**
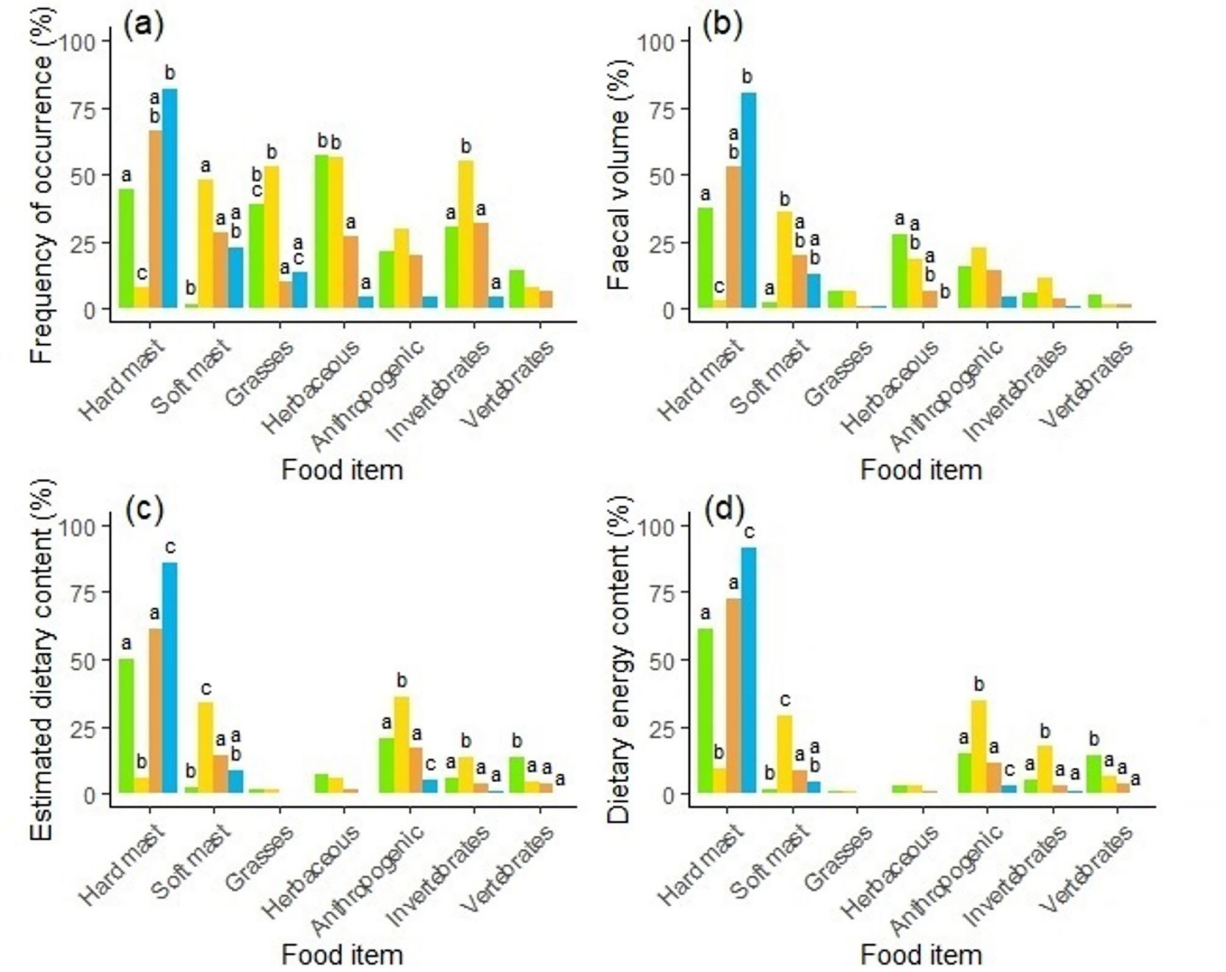
Seasonal variation in brown bear diet in the Western Carpathians, Slovakia, during 2013–2014. Shown are: (**a**) Frequency of occurrence (FO), (**b**) Faecal volume (FV), (**c**) Estimated dietary content (EDC), and (**d**) Estimated dietary energy content (EDEC) of major food items in spring (green), summer (yellow), autumn (orange), and winter (blue). Different letters above bars indicate significant differences among seasons within each food item (Tukey HSD test, *P* ≤ 0.05); bars sharing the same letter are not significantly different.

Our results showed that vegetation was the primary component of the brown bear diet, but the frequency of occurrence (Fig. 2a) and faecal volume (Fig. 2b) of food items, particularly highly nutritious types, varied significantly by season (Fig. 2). Although bears consumed herbaceous plants most frequently in spring (*FO* = 57%), hard mast had the highest FV (38%) and EDC (50%; Fig. 2c) and contributed with 61% of the total EDEC (Fig. 2d), followed by anthropogenic food (*EDEC* = 15%) and vertebrates (*EDEC* = 14%; Fig. 2d). In summer, bears frequently consumed herbaceous plans (*FO* = 57%), invertebrates (*FO* = 55%), grasses (*FO* = 53%) and soft mast (*FO* = 48%), however, soft mast (*FV* = 36%) and anthropogenic food (FV = 23%) were primary dietary items in term of volume, while hard mast decreased (*FO* = 8%, *FV* = 3%). However, in terms of contribution to EDEC, anthropogenic food was the primary source of energy (*EDEC* = 34%), followed by soft mast (*EDEC* = 29%) and vertebrates (*EDEC* = 17%). In autumn, hard mast was consumed most frequently (*FO* = 67%), and at the highest volume (*FV* = 53%) and dietary content (*EDC* = 61%), contributing with 73% of total of EDEC, followed by anthropogenic food (*EDEC* = 11%) and soft mast (*EDEC* = 9%). In winter, bears consumed hard mast most frequently (*FO* = 82%) and at the highest volume (*FV* = 80%) from all seasons, with contribution to the overall EDEC by 92%, however, our results may have been influenced by the low sample size (*n* = 22).

**Fig. 3:**
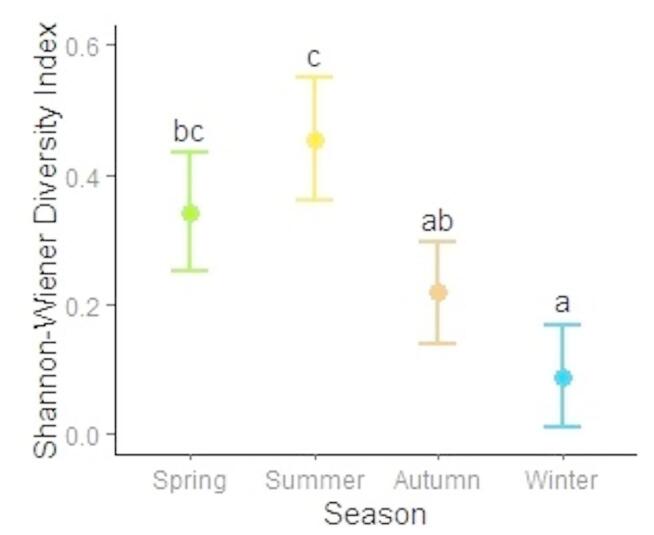
Seasonal Shannon-Wiener Diversity Index (*H*’; mean ± CI95%) of brown bear diet in the Western Carpathians, Slovakia during 2013–2014. Different letters indicate significant differences in diet diversity (Tukey HSD test, *P* ≤ 0.05); seasons sharing the same letter are not significantly different.

Shannon-Wiener diversity index varied significantly across seasons, with the highest diversity observed in summer (*H*’ = 0.45) and the lowest in winter (*H*’ = 0.09). Spring and autumn exhibited intermediate values (*H*’ = 0.34 and *H*’ = 0.22, respectively), with spring diversity being similar to autumn but significantly higher than winter (Fig. 3).

### Sex-specific diet composition

**Fig. 4:**
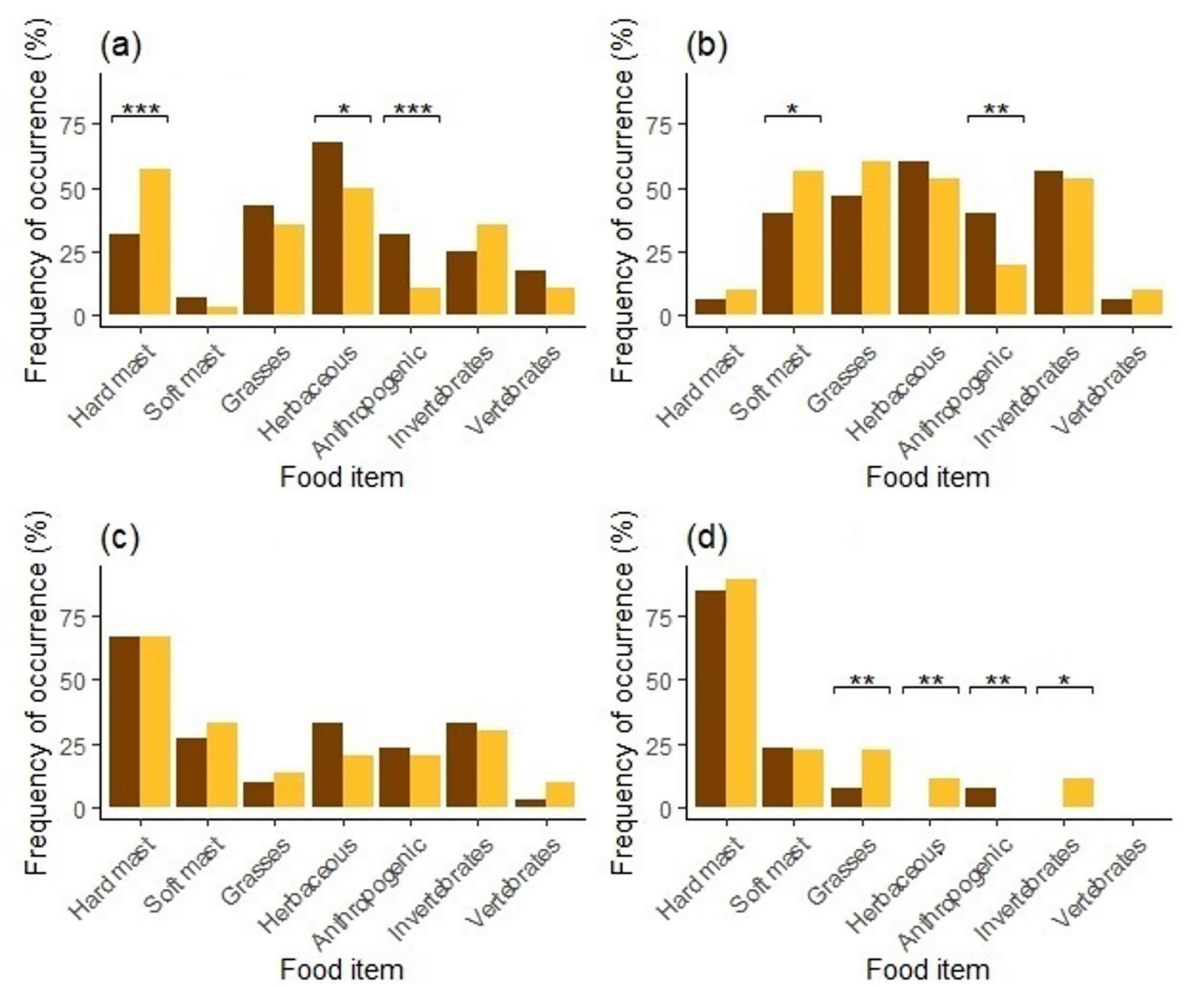
Frequency of occurrence (*FO*) of the main food items in diet of male (dark) and female (light) brown bears during spring (**a**), summer (**b**), autumn (**c**) and winter (**d**) in the Western Carpathians, Slovakia in 2013–2014. *Note* * *P* ≤ 0.05; ** *P* ≤ 0.01; *** *P* ≤ 0.001; no symbol = n.s.

**Fig. 5:**
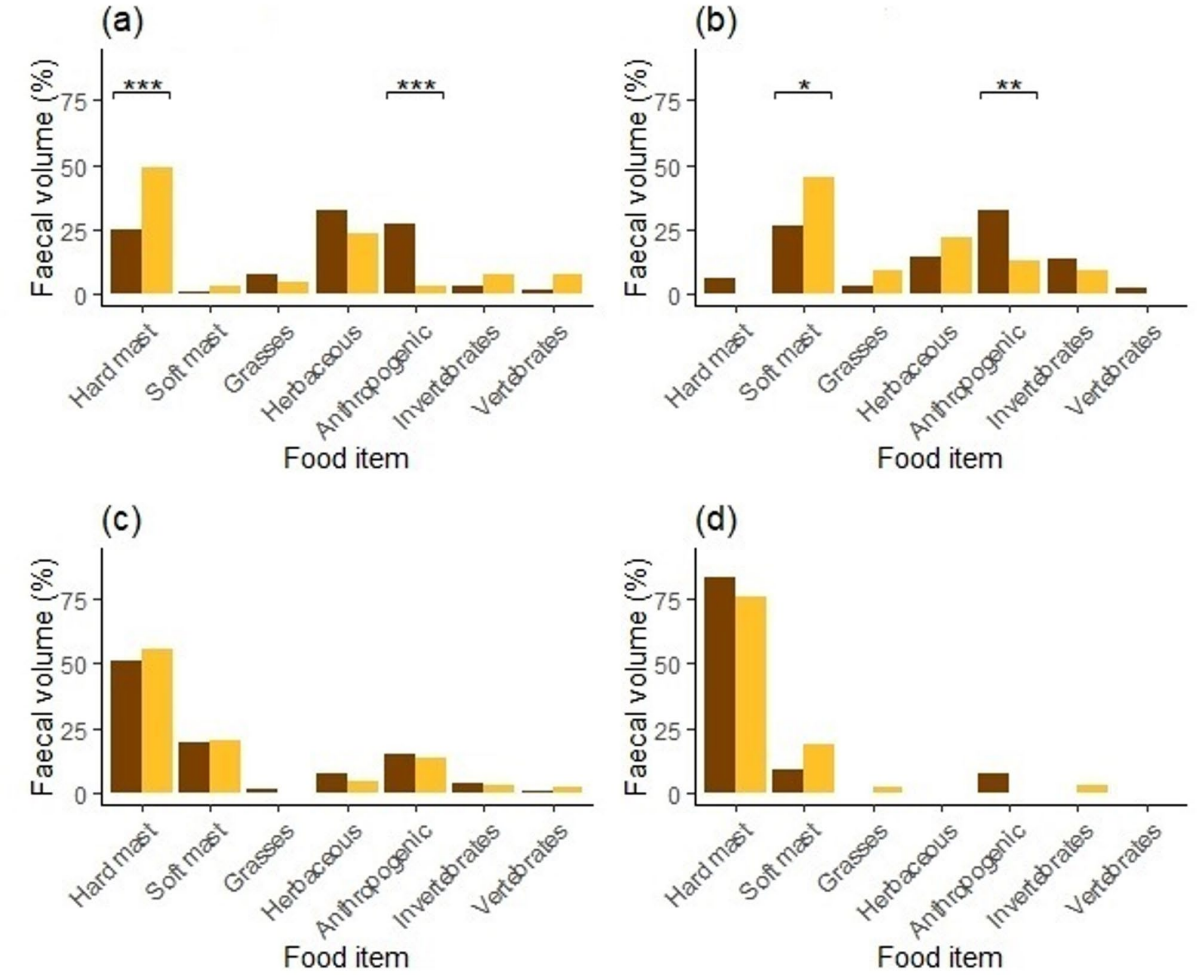
Faecal of volume (*FV*) of the main food items in diet of male (dark) and female (light) brown bears during spring (**a**), summer (**b**), autumn (**c**) and winter (**d**) in the Western Carpathians, Slovakia in 2013–2014. *Note* * *P* ≤ 0.05; ** *P* ≤ 0.01; *** *P* ≤ 0.001; no symbol = n.s.

**Fig. 6:**
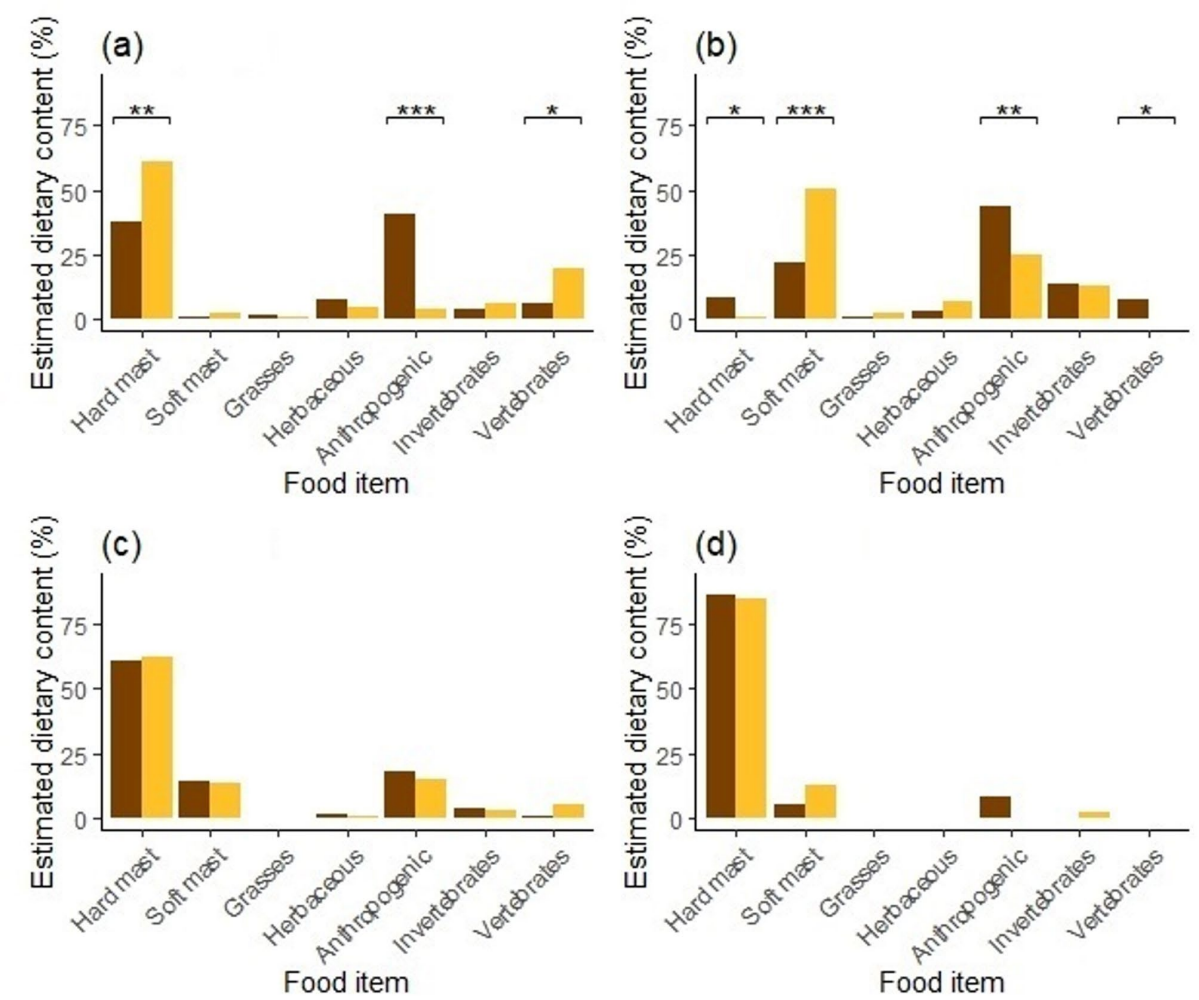
Estimated dietary content (EDC) of the main food items in diet of male (dark) and female (light) brown bears during spring (**a**), summer (**b**), autumn (**c**) and winter (**d**) in the Western Carpathians, Slovakia in 2013–2014. *Note* * *P* ≤ 0.05; ** *P* ≤ 0.01; *** *P* ≤ 0.001; no symbol = n.s.

Significant differences in diet composition between male and female brown bears were observed across all seasons (Fig. 4). In spring, females consumed hard mast more frequently than males (*FO* = 57% vs. *FO* = 32%; *χ*^2^ = 11.66, *P* ≤ 0.001), while males consumed herbaceous plants (*FO* = 68% vs. *FO* = 50%; *χ*^2^ = 5.90, *P* = 0.015) and anthropogenic food (*FO* = 32% vs. *FO* = 11%; *χ*^2^ = 12.37, *P* ≤ 0.001) more frequently than females (Fig. 4). In summer, females consumed soft mast more frequently than males (*FO* = 57% vs. *FO* = 40%; *χ*^2^ = 4.94, *P* = 0.026), while males consumed anthropogenic food more frequently than females (*FO* = 40% vs. *FO* = 20%; *χ*^2^ = 8.60, *P* = 0.003). In winter, females consumed grasses (*FO* = 22% vs. *FO* = 8%; *χ*^2^ = 7.17, *P* = 0.007), herbaceous plants (*FO* = 11% vs. *FO* = 0%; *χ*^2^ = 9.73, *P* = 0.002), and invertebrates (*FO* = 11% vs. *FO* = 0%; *χ*^2^ = 9.73, *P* = 0.002) more often than males, while males consumed anthropogenic food more frequent than females (*FO* = 8% vs. *FO* = 0%; *χ*^2^ = 6.06, *P* = 0.014; Fig. 4d). Seasonal differences in faecal volume mirrored these patterns (Fig. 5), with females consuming more hard mast in spring (*FV* = 49% vs. 25%; *χ*^2^ = 11.20, *P* ≤ 0.001) and more soft mast in summer (*FV* = 46% vs. 27%; *χ*^2^ = 4.94, *P* = 0.026). Males consumed more anthropogenic food across all seasons, particularly in spring (*FV* = 27% vs. 4%; *χ*^2^ = 19.95, *P* ≤ 0.001) and summer (*FV* = 32% vs. 13%; *χ*^2^ = 9.29, *P* = 0.002). No significant differences were found between male and female bears in autumn for *FO* (Fig. 4), *FV* (Fig. 5), or *EDC* (Fig. 6).

**Fig. 7:**
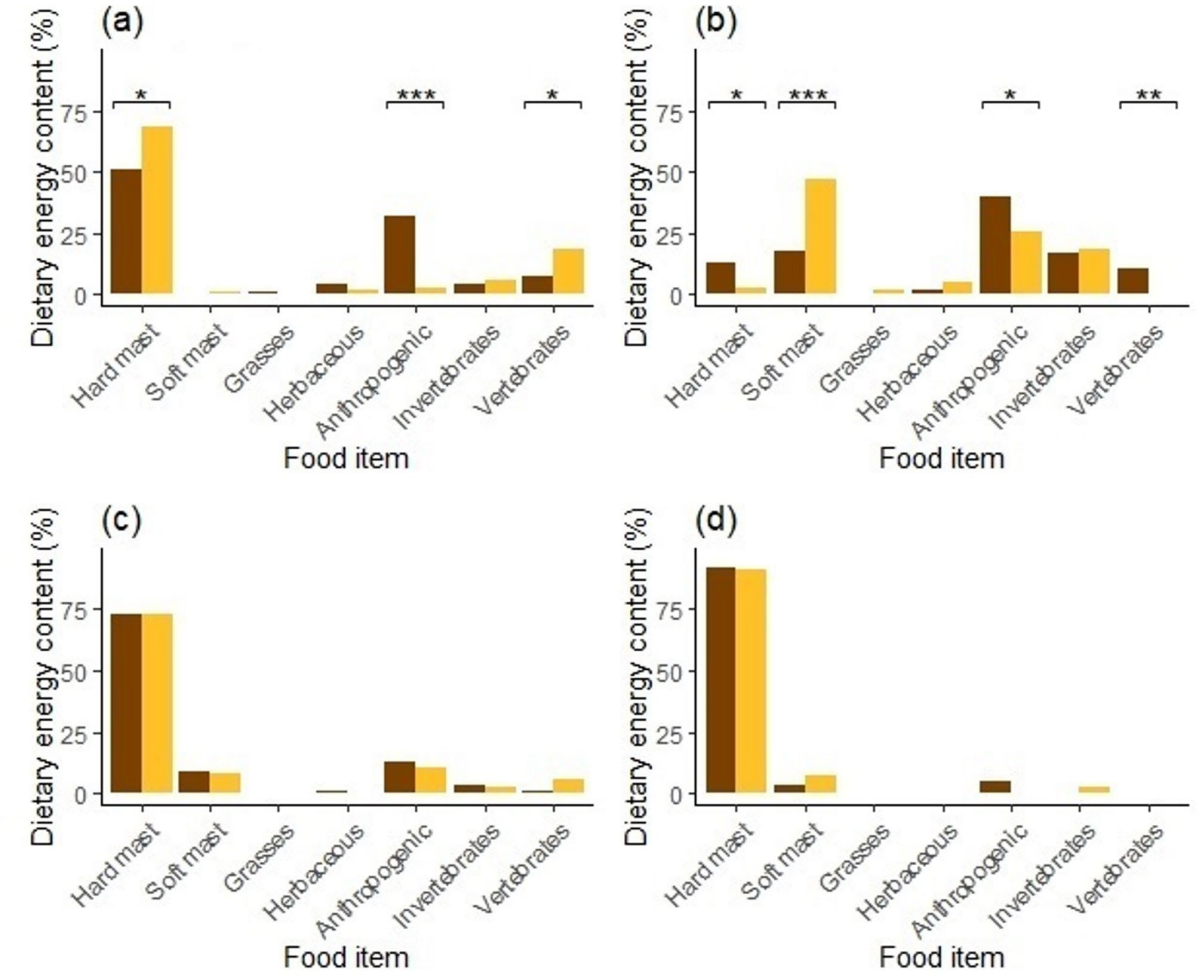
Estimated dietary energy content (EDEC) of the main food items in diet of male (dark) and female (light) brown bears during spring (**a**), summer (**b**), autumn (**c**) and winter (**d**) in the Western Carpathians, Slovakia in 2013–2014. *Note* * *P* ≤ 0.05; ** *P* ≤ 0.01; *** *P* ≤ 0.001; no symbol = n.s.

In summary, females gained significantly more energy from hard mast (*EDEC* = 68% vs. *EDEC* = 51%; *χ*^2^ = 5.66, *P* = 0.017) and vertebrates (*EDEC* = 19% vs. *EDEC* = 7%; *χ*^2^ = 4.83, *P* = 0.023) in spring, while males in contrast gained more energy from anthropogenic food (*EDEC* = 32% vs. *EDEC* = 3%; *χ*^2^ = 27.14, *P* ≤ 0.001; Fig. 7). In summer, females gained more energy from soft mast (*EDEC* = 47% vs. *EDEC* = 18%; *χ*^2^ = 17.99, *P* ≤ 0.001), while males obtained more energy from anthropogenic food (*EDEC* = 40% vs. *EDEC* = 26%; *χ*^2^ = 3.866, *P* = 0.050), hard mast (*EDEC* = 13% vs. *EDEC* = 2%; *χ*^2^ = 6.58, *P* = 0.010), and vertebrates (*EDEC* = 10% vs. *EDEC* = 0.3%; *χ*^2^ = 8.18, *P* = 0.004). No statistical differences in EDEC% were found between males and females in autumn or winter (Fig. 7).

No statistical differences were observed in the seasonal Shannon-Wiener Diversity Index between males and females (*LME*: *F* = 0.005, *P* = 0.939). Pianka’s index of niche overlap was highest in autumn (*O* = 0.98) and winter (*O* = 0.93) but decreased in spring (*O* = 0.72) and summer (*O* = 0.78).

## Discussion

Our results confirm that vegetation constituted the primary component of the brown bear diet across all seasons, consistent with our initial hypothesis and aligning with findings from other European brown bear populations^3,5,6,47–50^. Nonetheless, diet composition exhibited significant seasonal variation. Hard mast dominated bear diet in spring, autumn, and winter, whereas soft mast and anthropogenic food were predominant in summer. In terms of energetic contribution, hard mast represented the primary source across most seasons, except in summer, when anthropogenic food accounted for the highest proportion of dietary energy intake (34%). We also found significant sex-related differences in dietary composition, particularly in relation to anthropogenic food, which was consistently higher in males throughout the year. These differences were most pronounced during spring and summer, when males also consumed more vertebrate prey and hard mast, while females relied more on soft mast. Seasonal shifts in dietary diversity were evident, with the highest diversity observed in summer and the lowest in winter. Similarly, dietary niche overlap between sexes was lowest in spring and summer, indicating greater trophic differentiation during these periods. Collectively, these findings underscore the dietary flexibility of brown bears in a human-modified landscape and emphasize the role of seasonal resource availability in shaping their foraging strategies^4^.

Brown bear dietary patterns varied seasonally, however, a strong dependence on energy intake from hard mast was evident in spring (61%), autumn (73%), and winter (92%), with the lowest contribution recorded in summer (9%). Hard mast, particularly from beech and oak, provides a critical high-energy food source that enables bears to accumulate fat reserves during the later hyperphagic period (autumn) in preparation for hibernation^14,51^. Notably, our findings suggest that hard mast may serve as an important dietary component not only during hyperphagia but also during hibernation and the subsequent spring hypophagia, potentially supporting both energy conservation during hibernation and recovery post-hibernation. The low dietary diversity observed in winter, together with the overwhelming dominance of hard mast, indicates that Carpathian bears optimize energy intake during harsh conditions by foraging on this high-caloric resource. Similar winter foraging patterns have been reported in nearby bear populations in central^6^ and eastern Slovakia^5^. However, while our summer and autumn values were comparable to those studies, our spring and winter estimates were substantially higher, i.e. 61% and 92%, respectively, compared to 13% and 24% in spring, and 54% and 59% in winter^5,6^. These differences are likely due to spatial and temporal variation in hard mast availability, which follows locally-specific cycles driven by forest dynamics^52^. Nevertheless, the predominance of hard mast in brown bear diets during spring, autumn, and winter has also been reported from other European populations, including those in Croatia^47^, Spain^48^, Italy^3^, and Greece^50^, highlighting the broad ecological importance of this resource across the species’ range.

In contrast to other seasons, the summer diet of brown bears was the most diverse (Fig. 3), with soft mast, anthropogenic food, and invertebrates contributing substantially to dietary energy intake (Fig. 2d). This pattern is consistent with observations from other Slovak populations^5,6^, as well as those from Croatia^47^, Italy^3^, and Greece^50^. Although soft mast is typically low in protein^53^, it constitutes a valuable source of readily digestible carbohydrates that fulfil bears’ immediate energy demands during the active summer period^51^. In addition, bears relied on anthropogenic food sources for energy, particularly in summer (34%) and to a lesser extent in spring (14%). Similar to soft mast, anthropogenic foods such as agricultural crops or waste are rich in carbohydrates and represent a highly accessible energy supplement^51^. However, increased exploitation of these human-derived resources may alter natural foraging behaviour and elevate the risk of human-wildlife conflict^4^. The availability of anthropogenic food is driven by human activities, such as crop cultivation in summer and supplementary feeding of wild ungulates in winter, which create highly attractive foraging opportunities^5,6^, which can alter bear behaviour and compromise their function as a keystone species, potentially affecting trophic interactions. Access to high-caloric anthropogenic food has been linked to increased reproductive success^18^, potentially contributing to bear population growth in human-dominated landscapes. Comparable levels of energy intake from anthropogenic food have been reported in the nearby Polana Mountains (22% in summer and 12% in winter)^6^. In contrast, bears in eastern Slovakia derived the greatest proportion of their energy from anthropogenic sources in spring (60%), followed by summer (41%), winter (39%), and autumn (6%)^5^, suggesting regional variability in both availability and use of these resources. Invertebrates contributed to energy intake primarily in spring (14%) and summer (17%), although these levels were lower than those observed in Slovenia, where insects provided up to 50% of dietary energy^4^. Wasps/bees and ants were the predominant invertebrate taxa consumed (Tab. S1), a pattern consistent with findings from other Slovak^5,6^ and European bear populations^47,54^. Additionally, the increased consumption of ungulate carcasses in spring may reflect opportunistic scavenging of winter mortality or kleptoparasitism, i.e., the use of kills made by other predators^55^.

We found significant differences in dietary habits of male and female bears during spring and summer (Fig. 7). Notably, males consistently consumed higher quantities of anthropogenic food across all seasons, with the most pronounced differences occurring in spring and summer. In spring, females gained significantly more energy from natural food sources such as hard mast and vertebrates, whereas males relied heavily on anthropogenic food. This dietary pattern in females is likely associated with the increased protein requirements for lean mass accumulation and lactation^56^, combined with the need to avoid direct competition with larger, dominant males^57^. In summer, males continued to obtain more energy from anthropogenic sources, but also consumed greater amounts of hard mast and vertebrates, while females primarily relied on soft mast. These patterns align with findings in other bear species, where sexual differences in diet have been linked to sexual size dimorphism and corresponding variation in energetic demands and foraging efficiency. For example, sex-specific dietary divergence has been documented in grizzly bears (*Ursus arctos*)^57–59^, American black bears (*Ursus americanus*)^60^, Asiatic black bears (*Ursus thibetanus*)^2,61^, Himalayan brown bears (*Ursus arctos isabellinus*)^22^, and polar bears (*Ursus maritimus*)^62^. These studies consistently show that males tend to consume more animal-based foods, likely to meet higher energy demands associated with their larger body size and greater mobility. In human-modified landscapes, however, our findings suggest that males may substitute less accessible, high-caloric natural foods (e.g., large vertebrates) with more predictable and easily accessible anthropogenic resources with important ecological and physiological implications. In contrast, females, particularly those with dependent cubs, may avoid anthropogenic food sources to reduce the risk of intraspecific conflict and infanticide^61,63^. We suggest that these dietary differences are shaped by a combination of energetic optimization strategies and risk avoidance behaviours, reflecting sex-specific trade-offs in resource selection.

### Implications for bear management

Our findings have several important implications for the conservation and management of brown bears in human-dominated landscapes of Europe. First, the substantial contribution of anthropogenic food sources, particularly during spring (via supplemental feeding) and summer (through consumption of agricultural crops), underscores the necessity for proactive strategies to mitigate human-bear conflicts. A more regulated and spatially targeted approach to supplemental feeding is critical to reduce the risk of habituation and to limit the escalation of conflict incidents, including bear attacks on humans^64^. Management interventions should prioritize reducing bear access to anthropogenic food by securing waste containers, removing food remnants from recreational areas, and installing bear-proof fencing around high-conflict agricultural zones. Nevertheless, human-bear conflicts are complex, involving not only crop damage but also livestock predation and public safety concerns. The pronounced sex-specific patterns in anthropogenic food use, where males consistently exhibited higher reliance on human-derived foods, suggest that management frameworks should incorporate behavioural and demographic heterogeneity. For instance, conflict prevention efforts might be optimized by intensifying mitigation measures in male-dominated habitats, especially during periods of elevated anthropogenic food availability in spring and summer. The observed dietary plasticity and seasonal niche shifts reflect the adaptive foraging strategies of brown bears in response to fluctuating resource availability. However, this flexibility may entail ecological costs if it results in long-term substitution of natural foods with anthropogenic subsidies. Continuous, long-term monitoring of bear diet composition and habitat use is therefore essential to evaluate the sustainability of current foraging behaviours and to inform adaptive management strategies^65^. Finally, consistent with previous research in Slovakia^5,6^, we found no evidence of domestic livestock remains in the analysed bear scats, challenging prevailing assumptions regarding the livestock predation. Fostering human-bear coexistence in shared landscapes will require an integrative management approach that aligns the ecological requirements of brown bears with human land-use practices, grounded in evidence-based policy and active stakeholder engagement.

## Supplementary Information

Below is the link to the electronic supplementary material.

Supplementary Material 1

## Data Availability

Data generated by this study are available in the Supplementary material (Table S2).
