# Supplementary material for: Sex specific seasonal variation in the diet of brown bears in human dominated landscapes
Source: Sci Rep. 2025 Oct 27;15:37400. doi: 10.1038/s41598-025-21232-x (PMC12559401; doi:10.1038/s41598-025-21232-x)
Supplement: Supplementary file 1 — Supplementary Material 1 [file 41598_2025_21232_MOESM1_ESM.docx]

SUPPLEMENTARY MATERIAL

Table S1. Diet composition of brown bear males and females during 2013–2014 in the Western Carpathians, Slovakia.

| **Food category (male)** | **CF1** | **CF2** | **Spring** | | | | **Summer** | | | | **Autumn** | | | | **Winter** | | | |
| --- | --- | --- | --- | --- | --- | --- | --- | --- | --- | --- | --- | --- | --- | --- | --- | --- | --- | --- |
|  |  |  | **FO** | **FV** | **EDC** | **EDEC** | **FO** | **FV** | **EDC** | **EDEC** | **FO** | **FV** | **EDC** | **EDEC** | **FO** | **FV** | **EDC** | **EDEC** |
| Hard mast | 1.5 | 22.7 | 32.1 | 25.4 | 37.2 | 50.9 | 6.7 | 6.2 | 8.3 | 13.1 | 66.7 | 50.9 | 60.6 | 72.9 | 84.6 | 83.2 | 86.5 | 92.0 |
| Soft mast | 0.9 | 11.7 | 7.1 | 1.2 | 1.1 | 0.7 | 40.0 | 26.9 | 21.8 | 17.6 | 26.7 | 19.6 | 14.0 | 8.7 | 23.1 | 8.9 | 5.5 | 3.0 |
| Grasses | 0.3 | 7.4 | 42.9 | 7.9 | 1.9 | 0.9 | 46.7 | 3.5 | 0.8 | 0.4 | 10.0 | 1.4 | 0.3 | 0.1 | 7.7 | 0.2 | 0.0 | 0.0 |
| Herbaceous | 0.3 | 7.4 | 67.9 | 32.3 | 9.5 | 4.2 | 60.0 | 14.4 | 3.9 | 2.0 | 33.3 | 7.9 | 1.9 | 0.7 | 0.0 | 0.0 | 0.0 | 0.0 |
| Anthropogenic | 1.5 | 13.2 | 32.1 | 27.3 | 40.0 | 31.8 | 40.0 | 32.4 | 43.7 | 39.9 | 23.3 | 15.4 | 18.4 | 12.9 | 7.7 | 7.7 | 8.0 | 4.9 |
| Vertebrates | - | - | 17.9 | - | - | - | 6.7 | - | - | - | 3.3 | - | - | - | 0.0 | - | - | - |
| Ungulates | 3.0 | 19.3 | - | 2.2 | 6.4 | 7.4 | - | 2.9 | 7.8 | 10.3 |  | 0.5 | 1.2 | 1.2 | - | 0.0 | 0.0 | 0.0 |
| Rodents | 4.0 | 18.8 | - | 0.0 | 0.0 | 0.0 | - | 0.0 | 0.1 | 0.1 |  | 0.0 | 0.0 | 0.0 | - | 0.0 | 0.0 | 0.0 |
| Invertebrates | - | - | 28.6 | - | - | - | 56.7 | - | - | - | 33.3 | - | - | - | 0.0 | - | - | - |
| *Hymenoptera* | 1.1 | 17.7 | - | 3.7 | 4.0 | 4.2 | - | 13.8 | 13.6 | 16.7 | - | 4.2 | 3.7 | 3.4 | - | 0.0 | 0.0 | 0.0 |
| *Coleoptera* | 1.1 | 11.3 | - | 0.0 | 0.0 | 0.0 | - | 0.0 | 0.0 | 0.0 | - | 0.0 | 0.0 | 0.0 | - | 0.0 | 0.0 | 0.0 |
|  |  |  |  |  |  |  |  |  |  |  |  |  |  |  |  |  |  |  |
| **Food category (female)** | **CF1** | **CF2** | **Spring** | | | | **Summer** | | | | **Autumn** | | | | **Winter** | | | |
|  |  |  | **FO** | **FV** | **EDC** | **EDEC** | **FO** | **FV** | **EDC** | **EDEC** | **FO** | **FV** | **EDC** | **EDEC** | **FO** | **FV** | **EDC** | **EDEC** |
| Hard mast | 1.5 | 22.7 | 57.1 | 49.3 | 60.2 | 68.4 | 10.0 | 0.7 | 1.3 | 2.4 | 66.7 | 55.2 | 61.8 | 72.8 | 88.9 | 76.0 | 84.5 | 90.8 |
| Soft mast | 0.9 | 11.7 | 3.6 | 3.6 | 2.6 | 1.5 | 56.7 | 45.0 | 49.9 | 46.6 | 33.3 | 20.6 | 13.8 | 8.4 | 22.2 | 18.8 | 12.6 | 7.0 |
| Grasses | 0.3 | 7.4 | 35.7 | 5.0 | 1.0 | 0.4 | 60.0 | 9.4 | 2.9 | 1.7 | 13.3 | 0.3 | 0.1 | 0.0 | 22.2 | 2.0 | 0.4 | 0.1 |
| Herbaceous | 0.3 | 7.4 | 50.0 | 23.2 | 5.7 | 2.1 | 53.3 | 22.0 | 8.2 | 4.8 | 20.0 | 4.7 | 1.1 | 0.4 | 11.1 | 0.0 | 0.0 | 0.0 |
| Anthropogenic | 1.5 | 13.2 | 10.7 | 3.5 | 4.3 | 2.9 | 20.0 | 13.3 | 24.6 | 26.0 | 20.0 | 13.3 | 14.9 | 10.2 | 0.0 | 0.0 | 0.0 | 0.0 |
| Vertebrates | - | - | 10.7 | - | - | - | 10.0 | - | - | - | 10.0 | - | - | - | 0.0 | - | - | - |
| Ungulates | 3.0 | 19.3 | - | 8.0 | 19.5 | 18.9 | - | 0.0 | 0.1 | 0.1 | - | 2.5 | 5.6 | 5.6 | - | 0.0 | 0.0 | 0.0 |
| Rodents | 4.0 | 18.8 | - | 0.0 | 0.0 | 0.0 | - | 0.0 | 0.1 | 0.2 | - | 0.0 | 0.0 | 0.0 | - | 0.0 | 0.0 | 0.0 |
| Invertebrates | - | - | 35.7 | - | - | - | 56.7 | - | - | - | 33.3 | - | - | - | 11.1 | - | - | - |
| *Hymenoptera* | 1.1 | 17.7 | - | 7.5 | 6.7 | 5.9 | - | 9.6 | 13.0 | 18.3 | - | 3.4 | 2.8 | 2.6 | - | 3.1 | 2.5 | 2.1 |
| *Coleoptera* | 1.1 | 11.3 | - | 0.0 | 0.0 | 0.0 | - | 0.0 | 0.0 | 0.0 | - | 0.0 | 0.0 | 0.0 | - | 0.0 | 0.0 | 0.0 |

Table S2. Data on seasonal diet composition (FV%) of brown bear males (M) and females (F) during 2013–2014 in the Western Carpathians, Slovakia, used in our study.

| Sample_ID | Sex | Season | Hard mast | Soft mast | Grasses | Herbaceous | Anthropogen. | Invertebrates | Vertebrates |
| --- | --- | --- | --- | --- | --- | --- | --- | --- | --- |
| 1 | F | Spring | 0.00 | 0.00 | 0.00 | 100.00 | 0.00 | 0.00 | 0.00 |
| 2 | F | Spring | 99.49 | 0.00 | 0.00 | 0.51 | 0.00 | 0.00 | 0.00 |
| 3 | F | Spring | 99.43 | 0.00 | 0.00 | 0.00 | 0.00 | 0.57 | 0.00 |
| 4 | F | Spring | 0.00 | 0.00 | 0.00 | 96.53 | 2.97 | 0.50 | 0.00 |
| 5 | F | Spring | 1.10 | 0.00 | 5.49 | 31.10 | 0.00 | 2.85 | 59.46 |
| 6 | F | Spring | 0.00 | 0.00 | 13.03 | 2.30 | 0.00 | 0.00 | 84.67 |
| 7 | F | Spring | 85.00 | 0.00 | 15.00 | 0.00 | 0.00 | 0.00 | 0.00 |
| 8 | F | Spring | 0.00 | 0.00 | 0.00 | 19.35 | 0.00 | 80.65 | 0.00 |
| 9 | F | Spring | 100.00 | 0.00 | 0.00 | 0.00 | 0.00 | 0.00 | 0.00 |
| 10 | F | Spring | 100.00 | 0.00 | 0.00 | 0.00 | 0.00 | 0.00 | 0.00 |
| 11 | F | Spring | 100.00 | 0.00 | 0.00 | 0.00 | 0.00 | 0.00 | 0.00 |
| 12 | F | Spring | 97.01 | 0.00 | 2.99 | 0.00 | 0.00 | 0.00 | 0.00 |
| 13 | F | Spring | 50.00 | 0.00 | 0.00 | 50.00 | 0.00 | 0.00 | 0.00 |
| 14 | F | Spring | 100.00 | 0.00 | 0.00 | 0.00 | 0.00 | 0.00 | 0.00 |
| 15 | F | Spring | 0.00 | 0.00 | 1.89 | 61.32 | 0.00 | 36.79 | 0.00 |
| 16 | F | Spring | 100.00 | 0.00 | 0.00 | 0.00 | 0.00 | 0.00 | 0.00 |
| 17 | F | Spring | 0.00 | 0.00 | 2.99 | 97.01 | 0.00 | 0.00 | 0.00 |
| 18 | F | Spring | 0.00 | 99.49 | 0.00 | 0.00 | 0.00 | 0.51 | 0.00 |
| 19 | F | Spring | 0.00 | 0.00 | 11.30 | 59.24 | 0.00 | 29.46 | 0.00 |
| 20 | F | Spring | 100.00 | 0.00 | 0.00 | 0.00 | 0.00 | 0.00 | 0.00 |
| 21 | F | Spring | 0.00 | 0.00 | 0.79 | 3.14 | 12.47 | 3.83 | 79.77 |
| 22 | F | Spring | 100.00 | 0.00 | 0.00 | 0.00 | 0.00 | 0.00 | 0.00 |
| 23 | F | Spring | 48.08 | 0.00 | 48.08 | 3.85 | 0.00 | 0.00 | 0.00 |
| 24 | F | Spring | 100.00 | 0.00 | 0.00 | 0.00 | 0.00 | 0.00 | 0.00 |
| 25 | F | Spring | 100.00 | 0.00 | 0.00 | 0.00 | 0.00 | 0.00 | 0.00 |
| 26 | F | Spring | 0.00 | 0.00 | 0.00 | 0.00 | 83.65 | 16.35 | 0.00 |
| 27 | F | Spring | 0.00 | 0.00 | 0.00 | 62.50 | 0.00 | 37.50 | 0.00 |
| 28 | F | Spring | 0.00 | 0.00 | 37.50 | 62.50 | 0.00 | 0.00 | 0.00 |
| 29 | M | Spring | 0.00 | 0.00 | 0.00 | 0.00 | 100.00 | 0.00 | 0.00 |
| 30 | M | Spring | 0.00 | 0.00 | 14.56 | 82.52 | 0.00 | 2.91 | 0.00 |
| 31 | M | Spring | 0.00 | 0.00 | 100.00 | 0.00 | 0.00 | 0.00 | 0.00 |
| 32 | M | Spring | 0.00 | 0.00 | 0.00 | 0.00 | 100.00 | 0.00 | 0.00 |
| 33 | M | Spring | 85.00 | 0.00 | 0.00 | 0.00 | 0.00 | 0.00 | 15.00 |
| 34 | M | Spring | 100.00 | 0.00 | 0.00 | 0.00 | 0.00 | 0.00 | 0.00 |
| 35 | M | Spring | 100.00 | 0.00 | 0.00 | 0.00 | 0.00 | 0.00 | 0.00 |
| 36 | M | Spring | 84.93 | 0.00 | 0.00 | 0.00 | 0.00 | 0.00 | 15.07 |
| 37 | M | Spring | 56.86 | 0.00 | 2.01 | 2.01 | 0.00 | 39.13 | 0.00 |
| 38 | M | Spring | 0.00 | 0.00 | 2.00 | 2.00 | 64.87 | 15.57 | 15.57 |
| 39 | M | Spring | 96.78 | 0.00 | 3.22 | 0.00 | 0.00 | 0.00 | 0.00 |
| 40 | M | Spring | 0.00 | 0.00 | 2.55 | 82.83 | 0.00 | 0.00 | 14.62 |
| 41 | M | Spring | 0.00 | 0.00 | 50.00 | 50.00 | 0.00 | 0.00 | 0.00 |
| 42 | M | Spring | 0.00 | 0.00 | 14.56 | 82.52 | 0.00 | 2.91 | 0.00 |
| 43 | M | Spring | 0.00 | 0.00 | 0.00 | 2.99 | 97.01 | 0.00 | 0.00 |
| 44 | M | Spring | 0.00 | 0.00 | 0.00 | 0.00 | 99.49 | 0.51 | 0.00 |
| 45 | M | Spring | 0.00 | 23.08 | 0.00 | 38.46 | 0.00 | 38.46 | 0.00 |
| 46 | M | Spring | 14.49 | 0.00 | 2.90 | 82.13 | 0.00 | 0.48 | 0.00 |
| 47 | M | Spring | 80.65 | 0.00 | 0.00 | 19.35 | 0.00 | 0.00 | 0.00 |
| 48 | M | Spring | 93.41 | 0.00 | 3.30 | 3.30 | 0.00 | 0.00 | 0.00 |
| 49 | M | Spring | 0.00 | 0.00 | 0.00 | 100.00 | 0.00 | 0.00 | 0.00 |
| 50 | M | Spring | 0.00 | 0.00 | 15.00 | 85.00 | 0.00 | 0.00 | 0.00 |
| 51 | M | Spring | 0.00 | 0.00 | 0.00 | 100.00 | 0.00 | 0.00 | 0.00 |
| 52 | M | Spring | 0.00 | 10.44 | 10.44 | 39.56 | 39.56 | 0.00 | 0.00 |
| 53 | M | Spring | 0.00 | 0.00 | 0.00 | 15.94 | 83.55 | 0.00 | 0.51 |
| 54 | M | Spring | 0.00 | 0.00 | 0.00 | 15.00 | 85.00 | 0.00 | 0.00 |
| 55 | M | Spring | 0.00 | 0.00 | 0.00 | 97.03 | 0.00 | 2.97 | 0.00 |
| 56 | M | Spring | 0.00 | 0.00 | 0.00 | 4.35 | 95.65 | 0.00 | 0.00 |
| 57 | F | Summer | 0.00 | 0.00 | 1.98 | 0.00 | 98.02 | 0.00 | 0.00 |
| 58 | F | Summer | 0.00 | 0.00 | 28.09 | 28.09 | 0.00 | 43.82 | 0.00 |
| 59 | F | Summer | 1.98 | 24.75 | 24.75 | 9.90 | 0.00 | 38.61 | 0.00 |
| 60 | F | Summer | 0.00 | 0.00 | 0.00 | 100.00 | 0.00 | 0.00 | 0.00 |
| 61 | F | Summer | 0.00 | 0.00 | 34.06 | 34.06 | 0.00 | 31.88 | 0.00 |
| 62 | F | Summer | 0.00 | 0.00 | 16.28 | 21.77 | 0.00 | 61.52 | 0.43 |
| 63 | F | Summer | 0.00 | 80.41 | 1.40 | 1.40 | 0.00 | 16.58 | 0.21 |
| 64 | F | Summer | 0.00 | 1.88 | 0.00 | 0.00 | 61.02 | 36.61 | 0.49 |
| 65 | F | Summer | 0.00 | 94.20 | 0.00 | 2.90 | 0.00 | 2.90 | 0.00 |
| 66 | F | Summer | 0.00 | 59.06 | 0.00 | 0.00 | 37.97 | 2.98 | 0.00 |
| 67 | F | Summer | 0.00 | 100.00 | 0.00 | 0.00 | 0.00 | 0.00 | 0.00 |
| 68 | F | Summer | 0.00 | 96.53 | 2.97 | 0.00 | 0.00 | 0.50 | 0.00 |
| 69 | F | Summer | 0.00 | 81.13 | 3.77 | 0.00 | 15.09 | 0.00 | 0.00 |
| 70 | F | Summer | 0.00 | 99.49 | 0.00 | 0.00 | 0.00 | 0.51 | 0.00 |
| 71 | F | Summer | 0.00 | 100.00 | 0.00 | 0.00 | 0.00 | 0.00 | 0.00 |
| 72 | F | Summer | 15.94 | 83.57 | 0.00 | 0.00 | 0.00 | 0.48 | 0.00 |
| 73 | F | Summer | 0.00 | 94.20 | 2.90 | 0.00 | 0.00 | 2.90 | 0.00 |
| 74 | F | Summer | 0.00 | 94.19 | 2.90 | 0.00 | 0.00 | 2.91 | 0.00 |
| 75 | F | Summer | 0.00 | 100.00 | 0.00 | 0.00 | 0.00 | 0.00 | 0.00 |
| 76 | F | Summer | 0.00 | 93.41 | 3.30 | 3.30 | 0.00 | 0.00 | 0.00 |
| 77 | F | Summer | 0.00 | 51.52 | 24.24 | 24.24 | 0.00 | 0.00 | 0.00 |
| 78 | F | Summer | 0.00 | 0.00 | 14.56 | 82.52 | 0.00 | 2.91 | 0.00 |
| 79 | F | Summer | 0.00 | 0.00 | 0.00 | 63.21 | 0.00 | 36.79 | 0.00 |
| 80 | F | Summer | 0.00 | 94.20 | 0.00 | 2.90 | 0.00 | 2.90 | 0.00 |
| 81 | F | Summer | 0.00 | 0.00 | 0.00 | 100.00 | 0.00 | 0.00 | 0.00 |
| 82 | F | Summer | 0.00 | 0.00 | 0.51 | 99.49 | 0.00 | 0.00 | 0.00 |
| 83 | F | Summer | 0.00 | 0.00 | 2.90 | 0.00 | 94.19 | 2.91 | 0.00 |
| 84 | F | Summer | 0.00 | 0.00 | 62.50 | 37.50 | 0.00 | 0.00 | 0.00 |
| 85 | F | Summer | 3.30 | 0.00 | 3.30 | 0.00 | 93.41 | 0.00 | 0.00 |
| 86 | F | Summer | 0.00 | 0.00 | 50.00 | 50.00 | 0.00 | 0.00 | 0.00 |
| 87 | M | Summer | 0.00 | 92.75 | 3.01 | 3.01 | 0.00 | 1.23 | 0.00 |
| 88 | M | Summer | 0.00 | 5.99 | 2.49 | 2.49 | 0.00 | 3.05 | 85.98 |
| 89 | M | Summer | 0.00 | 0.00 | 0.00 | 2.99 | 97.01 | 0.00 | 0.00 |
| 90 | M | Summer | 0.00 | 0.00 | 0.00 | 0.00 | 85.00 | 15.00 | 0.00 |
| 91 | M | Summer | 0.00 | 0.00 | 0.00 | 31.99 | 53.32 | 14.68 | 0.00 |
| 92 | M | Summer | 0.00 | 37.31 | 0.00 | 0.00 | 0.00 | 62.19 | 0.50 |
| 93 | M | Summer | 0.00 | 0.00 | 37.86 | 62.14 | 0.00 | 0.00 | 0.00 |
| 94 | M | Summer | 0.00 | 0.00 | 0.00 | 0.00 | 100.00 | 0.00 | 0.00 |
| 95 | M | Summer | 0.00 | 84.48 | 0.65 | 0.00 | 0.00 | 14.87 | 0.00 |
| 96 | M | Summer | 100.00 | 0.00 | 0.00 | 0.00 | 0.00 | 0.00 | 0.00 |
| 97 | M | Summer | 0.00 | 0.00 | 11.83 | 11.83 | 53.25 | 23.08 | 0.00 |
| 98 | M | Summer | 0.00 | 59.59 | 2.42 | 2.42 | 0.00 | 35.57 | 0.00 |
| 99 | M | Summer | 0.00 | 41.44 | 0.00 | 16.57 | 41.44 | 0.55 | 0.00 |
| 100 | M | Summer | 0.00 | 0.00 | 0.00 | 0.00 | 100.00 | 0.00 | 0.00 |
| 101 | M | Summer | 0.00 | 99.49 | 0.00 | 0.51 | 0.00 | 0.00 | 0.00 |
| 102 | M | Summer | 0.00 | 0.00 | 15.00 | 85.00 | 0.00 | 0.00 | 0.00 |
| 103 | M | Summer | 0.00 | 97.01 | 2.99 | 0.00 | 0.00 | 0.00 | 0.00 |
| 104 | M | Summer | 0.00 | 0.00 | 0.00 | 0.00 | 100.00 | 0.00 | 0.00 |
| 105 | M | Summer | 0.00 | 0.00 | 0.00 | 15.00 | 85.00 | 0.00 | 0.00 |
| 106 | M | Summer | 0.00 | 0.00 | 0.00 | 0.00 | 100.00 | 0.00 | 0.00 |
| 107 | M | Summer | 0.00 | 0.00 | 2.90 | 0.00 | 94.20 | 2.90 | 0.00 |
| 108 | M | Summer | 0.00 | 0.00 | 0.90 | 17.40 | 0.00 | 81.70 | 0.00 |
| 109 | M | Summer | 0.00 | 84.63 | 0.43 | 0.00 | 0.00 | 14.93 | 0.00 |
| 110 | M | Summer | 0.00 | 100.00 | 0.00 | 0.00 | 0.00 | 0.00 | 0.00 |
| 111 | M | Summer | 0.00 | 80.19 | 0.63 | 2.98 | 0.00 | 16.20 | 0.00 |
| 112 | M | Summer | 0.00 | 0.00 | 4.79 | 92.02 | 0.00 | 3.19 | 0.00 |
| 113 | M | Summer | 0.00 | 23.66 | 0.00 | 39.43 | 0.00 | 36.91 | 0.00 |
| 114 | M | Summer | 0.00 | 0.00 | 19.16 | 31.45 | 0.00 | 49.39 | 0.00 |
| 115 | M | Summer | 0.00 | 0.00 | 0.00 | 0.32 | 62.30 | 37.38 | 0.00 |
| 116 | M | Summer | 85.00 | 0.00 | 0.00 | 15.00 | 0.00 | 0.00 | 0.00 |
| 117 | F | Autumn | 100.00 | 0.00 | 0.00 | 0.00 | 0.00 | 0.00 | 0.00 |
| 118 | F | Autumn | 100.00 | 0.00 | 0.00 | 0.00 | 0.00 | 0.00 | 0.00 |
| 119 | F | Autumn | 0.00 | 69.91 | 0.00 | 0.00 | 3.64 | 13.91 | 12.55 |
| 120 | F | Autumn | 98.98 | 0.00 | 0.00 | 0.00 | 0.00 | 0.51 | 0.51 |
| 121 | F | Autumn | 97.01 | 0.00 | 0.00 | 0.00 | 0.00 | 2.99 | 0.00 |
| 122 | F | Autumn | 96.69 | 0.00 | 0.00 | 0.00 | 0.00 | 3.31 | 0.00 |
| 123 | F | Autumn | 100.00 | 0.00 | 0.00 | 0.00 | 0.00 | 0.00 | 0.00 |
| 124 | F | Autumn | 0.00 | 99.42 | 0.58 | 0.00 | 0.00 | 0.00 | 0.00 |
| 125 | F | Autumn | 97.01 | 0.00 | 0.00 | 2.99 | 0.00 | 0.00 | 0.00 |
| 126 | F | Autumn | 99.49 | 0.00 | 0.00 | 0.00 | 0.00 | 0.51 | 0.00 |
| 127 | F | Autumn | 0.00 | 100.00 | 0.00 | 0.00 | 0.00 | 0.00 | 0.00 |
| 128 | F | Autumn | 1.63 | 46.20 | 0.00 | 20.38 | 0.00 | 31.79 | 0.00 |
| 129 | F | Autumn | 100.00 | 0.00 | 0.00 | 0.00 | 0.00 | 0.00 | 0.00 |
| 130 | F | Autumn | 100.00 | 0.00 | 0.00 | 0.00 | 0.00 | 0.00 | 0.00 |
| 131 | F | Autumn | 0.00 | 15.00 | 0.00 | 0.00 | 85.00 | 0.00 | 0.00 |
| 132 | F | Autumn | 85.00 | 0.00 | 0.00 | 0.00 | 0.00 | 15.00 | 0.00 |
| 133 | F | Autumn | 100.00 | 0.00 | 0.00 | 0.00 | 0.00 | 0.00 | 0.00 |
| 134 | F | Autumn | 0.00 | 37.10 | 1.23 | 0.00 | 0.00 | 0.00 | 61.67 |
| 135 | F | Autumn | 100.00 | 0.00 | 0.00 | 0.00 | 0.00 | 0.00 | 0.00 |
| 136 | F | Autumn | 100.00 | 0.00 | 0.00 | 0.00 | 0.00 | 0.00 | 0.00 |
| 137 | F | Autumn | 80.65 | 0.00 | 0.00 | 0.00 | 0.00 | 19.35 | 0.00 |
| 138 | F | Autumn | 11.35 | 64.29 | 0.00 | 11.35 | 0.00 | 13.01 | 0.00 |
| 139 | F | Autumn | 0.76 | 0.00 | 0.00 | 0.00 | 99.24 | 0.00 | 0.00 |
| 140 | F | Autumn | 0.00 | 2.99 | 0.00 | 0.00 | 97.01 | 0.00 | 0.00 |
| 141 | F | Autumn | 0.00 | 82.52 | 2.91 | 0.00 | 14.56 | 0.00 | 0.00 |
| 142 | F | Autumn | 0.00 | 0.00 | 0.00 | 0.00 | 100.00 | 0.00 | 0.00 |
| 143 | F | Autumn | 94.20 | 0.00 | 0.00 | 2.90 | 0.00 | 2.90 | 0.00 |
| 144 | F | Autumn | 0.00 | 100.00 | 0.00 | 0.00 | 0.00 | 0.00 | 0.00 |
| 145 | F | Autumn | 93.41 | 0.00 | 3.30 | 3.30 | 0.00 | 0.00 | 0.00 |
| 146 | F | Autumn | 0.00 | 0.00 | 0.00 | 100.00 | 0.00 | 0.00 | 0.00 |
| 147 | M | Autumn | 31.97 | 53.28 | 0.00 | 0.00 | 0.00 | 14.74 | 0.00 |
| 148 | M | Autumn | 96.53 | 0.00 | 0.00 | 2.97 | 0.00 | 0.50 | 0.00 |
| 149 | M | Autumn | 0.00 | 1.72 | 0.00 | 1.72 | 1.72 | 94.85 | 0.00 |
| 150 | M | Autumn | 93.41 | 0.00 | 3.30 | 3.30 | 0.00 | 0.00 | 0.00 |
| 151 | M | Autumn | 100.00 | 0.00 | 0.00 | 0.00 | 0.00 | 0.00 | 0.00 |
| 152 | M | Autumn | 50.00 | 0.00 | 0.00 | 0.00 | 50.00 | 0.00 | 0.00 |
| 153 | M | Autumn | 2.99 | 0.00 | 0.00 | 0.00 | 97.01 | 0.00 | 0.00 |
| 154 | M | Autumn | 100.00 | 0.00 | 0.00 | 0.00 | 0.00 | 0.00 | 0.00 |
| 155 | M | Autumn | 0.00 | 0.00 | 0.00 | 0.00 | 100.00 | 0.00 | 0.00 |
| 156 | M | Autumn | 0.00 | 100.00 | 0.00 | 0.00 | 0.00 | 0.00 | 0.00 |
| 157 | M | Autumn | 97.01 | 0.00 | 0.00 | 0.00 | 0.00 | 2.99 | 0.00 |
| 158 | M | Autumn | 0.00 | 0.00 | 0.00 | 0.00 | 100.00 | 0.00 | 0.00 |
| 159 | M | Autumn | 2.90 | 0.00 | 36.23 | 60.39 | 0.00 | 0.48 | 0.00 |
| 160 | M | Autumn | 94.20 | 0.00 | 0.00 | 2.90 | 0.00 | 2.90 | 0.00 |
| 161 | M | Autumn | 97.01 | 0.00 | 0.00 | 2.99 | 0.00 | 0.00 | 0.00 |
| 162 | M | Autumn | 99.49 | 0.00 | 0.00 | 0.00 | 0.00 | 0.51 | 0.00 |
| 163 | M | Autumn | 100.00 | 0.00 | 0.00 | 0.00 | 0.00 | 0.00 | 0.00 |
| 164 | M | Autumn | 0.00 | 97.01 | 0.00 | 2.99 | 0.00 | 0.00 | 0.00 |
| 165 | M | Autumn | 85.00 | 0.00 | 0.00 | 0.00 | 0.00 | 0.00 | 15.00 |
| 166 | M | Autumn | 82.17 | 0.00 | 0.00 | 0.00 | 14.50 | 3.33 | 0.00 |
| 167 | M | Autumn | 0.00 | 36.41 | 0.00 | 60.68 | 0.00 | 2.91 | 0.00 |
| 168 | M | Autumn | 100.00 | 0.00 | 0.00 | 0.00 | 0.00 | 0.00 | 0.00 |
| 169 | M | Autumn | 0.00 | 100.00 | 0.00 | 0.00 | 0.00 | 0.00 | 0.00 |
| 170 | M | Autumn | 100.00 | 0.00 | 0.00 | 0.00 | 0.00 | 0.00 | 0.00 |
| 171 | M | Autumn | 0.00 | 0.00 | 0.00 | 0.00 | 100.00 | 0.00 | 0.00 |
| 172 | M | Autumn | 0.00 | 100.00 | 0.00 | 0.00 | 0.00 | 0.00 | 0.00 |
| 173 | M | Autumn | 97.01 | 0.00 | 2.99 | 0.00 | 0.00 | 0.00 | 0.00 |
| 174 | M | Autumn | 0.87 | 0.00 | 0.00 | 99.13 | 0.00 | 0.00 | 0.00 |
| 175 | M | Autumn | 96.53 | 0.00 | 0.00 | 0.50 | 0.00 | 2.97 | 0.00 |
| 176 | M | Autumn | 0.00 | 100.00 | 0.00 | 0.00 | 0.00 | 0.00 | 0.00 |
| 177 | F | Winter | 85.00 | 0.00 | 15.00 | 0.00 | 0.00 | 0.00 | 0.00 |
| 178 | F | Winter | 100.00 | 0.00 | 0.00 | 0.00 | 0.00 | 0.00 | 0.00 |
| 179 | F | Winter | 100.00 | 0.00 | 0.00 | 0.00 | 0.00 | 0.00 | 0.00 |
| 180 | F | Winter | 2.14 | 69.54 | 0.00 | 0.36 | 0.00 | 27.96 | 0.00 |
| 181 | F | Winter | 0.00 | 100.00 | 0.00 | 0.00 | 0.00 | 0.00 | 0.00 |
| 182 | F | Winter | 100.00 | 0.00 | 0.00 | 0.00 | 0.00 | 0.00 | 0.00 |
| 183 | F | Winter | 100.00 | 0.00 | 0.00 | 0.00 | 0.00 | 0.00 | 0.00 |
| 184 | F | Winter | 97.01 | 0.00 | 2.99 | 0.00 | 0.00 | 0.00 | 0.00 |
| 185 | F | Winter | 100.00 | 0.00 | 0.00 | 0.00 | 0.00 | 0.00 | 0.00 |
| 186 | M | Winter | 100.00 | 0.00 | 0.00 | 0.00 | 0.00 | 0.00 | 0.00 |
| 187 | M | Winter | 100.00 | 0.00 | 0.00 | 0.00 | 0.00 | 0.00 | 0.00 |
| 188 | M | Winter | 100.00 | 0.00 | 0.00 | 0.00 | 0.00 | 0.00 | 0.00 |
| 189 | M | Winter | 100.00 | 0.00 | 0.00 | 0.00 | 0.00 | 0.00 | 0.00 |
| 190 | M | Winter | 0.00 | 97.01 | 2.99 | 0.00 | 0.00 | 0.00 | 0.00 |
| 191 | M | Winter | 100.00 | 0.00 | 0.00 | 0.00 | 0.00 | 0.00 | 0.00 |
| 192 | M | Winter | 85.00 | 15.00 | 0.00 | 0.00 | 0.00 | 0.00 | 0.00 |
| 193 | M | Winter | 100.00 | 0.00 | 0.00 | 0.00 | 0.00 | 0.00 | 0.00 |
| 194 | M | Winter | 100.00 | 0.00 | 0.00 | 0.00 | 0.00 | 0.00 | 0.00 |
| 195 | M | Winter | 97.01 | 2.99 | 0.00 | 0.00 | 0.00 | 0.00 | 0.00 |
| 196 | M | Winter | 0.00 | 0.00 | 0.00 | 0.00 | 100.00 | 0.00 | 0.00 |
| 197 | M | Winter | 100.00 | 0.00 | 0.00 | 0.00 | 0.00 | 0.00 | 0.00 |
| 198 | M | Winter | 100.00 | 0.00 | 0.00 | 0.00 | 0.00 | 0.00 | 0.00 |
